# Prähabilitation abseits spezialisierter Zentren: Ist Prähabilitation auch auf dem Land machbar?

**DOI:** 10.1007/s00104-025-02295-y

**Published:** 2025-05-06

**Authors:** Steffen Seyfried, Christoph Reißfelder, Julia Hardt

**Affiliations:** Chirurgische Klinik, Universitätsmedizin Mannheim, Medizinische Fakultät Mannheim, Universität Heidelberg, Theodor Kutzer Ufer 1-3, 68167 Mannheim, Deutschland

**Keywords:** Perioperatives Management, Chirurgie, Telemedizin, Rehabilitation, Finanzierung, Perioperative management, Surgery, Telemedicine, Rehabilitation, Funding

## Abstract

Die Prähabilitation gewinnt zunehmend an Bedeutung als integraler Bestandteil des perioperativen Managements. Sie verbessert funktionelle Reserven vor chirurgischen Eingriffen und reduziert postoperative Komplikationen. Während spezialisierte Zentren erfolgreich Prähabilitationsprogramme implementieren, bleibt die flächendeckende Umsetzung, insbesondere in ländlichen Regionen, eine Herausforderung. Organisatorische, infrastrukturelle und finanzielle Hürden erschweren die Adaption außerhalb universitärer Einrichtungen. Digitale und hybride Ansätze bieten jedoch Potenzial, um eine wohnortnahe Versorgung zu ermöglichen. Eine nachhaltige Integration in das Gesundheitssystem erfordert sektorenübergreifende Kooperationen, politische Unterstützung und angepasste Vergütungsstrukturen.

## Abstract (en)

Prehabilitation is becoming increasingly more important as an integral component of perioperative management. It improves the functional reserves before surgical interventions and reduces postoperative complications. While specialized centers have successfully implemented prehabilitation programs, the comprehensive implementation especially in rural areas remains challenging. Organizational, infrastructural and financial barriers exacerbate the adaptation outside university facilities; however, hybrid and digital approaches offer promising solutions for decentralized care. A sustainable integration into the healthcare system requires interdisciplinary collaboration, political support and adapted reimbursement structures.

Die Prähabilitation entwickelt sich zu einem Schlüsselelement des perioperativen Managements. Während spezialisierte Zentren ihre Vorteile unter Beweis gestellt haben, bleibt eine breitere Umsetzung eine Herausforderung. Die Integration digitaler und hybrider Modelle könnte die Zugänglichkeit in nichtspezialisierten und ländlichen Umgebungen ermöglichen. Allerdings müssen finanzielle, logistische und infrastrukturelle Hürden überwunden werden, um die Prähabilitation als Behandlungsstandard zu etablieren.

## Hintergrund

Mit der zunehmenden Bekanntheit und Evidenz der Prähabilitation nutzen immer mehr medizinische Zentren und Kliniken diese Maßnahmen auch als ein Alleinstellungsmerkmal und zur Positionierung im Wettbewerb. Die Unterscheidung zwischen einer strukturierten Prähabilitation und einer allgemein perioperativen Betreuung ist dabei nicht immer klar abzugrenzen, da viele Elemente einer umfassenden präoperativen Patientenbetreuung ähnliche Ziele verfolgen [19]. Die Übergänge zwischen diesen Konzepten sind fließend, weshalb es nicht überraschend ist, dass Prähabilitationsmaßnahmen mittlerweile auch in die S3-Leitlinie Perioperatives Management bei gastrointestinalen Tumoren (POMGAT; [25]) aufgenommen wurden. Die Leitlinie betont die Bedeutung eines strukturierten perioperativen Managements für die Verbesserung des Behandlungsergebnisses von Patient:innen mit malignen Erkrankungen des Abdomens. Dabei wird die Prähabilitation als essenzieller Bestandteil dieses multimodalen Ansatzes genannt, insbesondere im Hinblick auf die Optimierung funktioneller Reserven vor einer anstehenden Operation. Dies unterstreicht, dass prähabilitative Maßnahmen nicht nur eine Ergänzung bestehender perioperativer Betreuungskonzepte darstellen, sondern zunehmend als standardisierte Empfehlung Einzug in die klinische Praxis halten [23].

Auch im Rahmen von Zertifizierungsprogrammen für onkologische Zentren werden zunehmend präoperative Maßnahmen als relevante Qualitätsindikatoren betrachtet. So sind bereits etablierte Programme wie das Patient Blood Management (PBM; [18]), dass eine gezielte Optimierung des Blutbildes vor Operationen beinhaltet, oder die psychoonkologische Betreuung, die oft bereits im präoperativen Zeitraum beginnt, integraler Bestandteil einer ganzheitlichen Patientenversorgung. Beide Konzepte lassen sich unter dem Dach der Prähabilitation subsumieren, da sie auf die präoperative Optimierung der Patient:innen abzielen.

Darüber hinaus gibt es mit den Enhanced-Recovery-After-Surgery(ERAS®)- und Fast-Track-Prinzipien bereits etablierte Programme zur funktionellen Wiederherstellung, die auch präoperative Elemente enthalten [15, 21]. Diese Konzepte zielen darauf ab, postoperative Komplikationen zu minimieren, die Mobilisierung zu beschleunigen und die Krankenhausverweildauer zu reduzieren. Auch hier gibt es klare Parallelen zur Prähabilitation, insbesondere in der Förderung körperlicher Aktivität und der Ernährungsoptimierung bereits vor der Operation.

Die Organisation der Implementierung von Prähabilitation ist komplex

Trotz dieser vielversprechenden Ergebnisse und der potenziellen Einsparungen im Gesundheitswesen bleibt die Implementierung prähabilitativer Programme in der klinischen Praxis eine Herausforderung. Dies liegt vor allem an der organisatorischen Komplexität sowie dem Bedarf an zusätzlichen finanziellen und personellen Ressourcen.

Angesichts dieser Entwicklungen wird deutlich, dass die Prähabilitation zunehmend als integraler Bestandteil eines umfassenden perioperativen Managements betrachtet wird. Die systematische Einbindung in Leitlinien und Zertifizierungsprozesse zeigt, dass es sich nicht mehr nur um ein optionales Zusatzangebot handelt, sondern um eine evidenzbasierte Strategie zur Verbesserung der perioperativen Ergebnisse.

Dies wirft die zentrale Frage auf: Ist Prähabilitation ein Konzept, das nur in hochspezialisierten Zentren realisierbar ist, oder gibt es Potenzial für eine flächendeckende Implementierung auch in peripheren und ländlichen Gebieten?

## Stand der Implementierung und Erfahrungen aus Zentren

Ein Blick auf die verfügbaren Studien zur Prähabilitation bei abdominalchirurgischen Eingriffen (Tab. 1) zeigt ein klares Muster: Nahezu alle existierenden Untersuchungen wurden in hochspezialisierten Zentren durchgeführt und sind als randomisierte kontrollierte Studien (RCTs) angelegt. Dies verdeutlicht, dass die Prähabilitation bislang fast ausschließlich in einem wissenschaftlich kontrollierten Umfeld getestet wurde, was eine breite Implementierung in der klinischen Routine erheblich erschwert.

**Tab. 1:** Übersicht über Studien zur Prähabilitation in der abdominellen Chirurgie

Autor	Studienart	Land	Supervision	Patientenzahl	Publikationsjahr
Pesce et al. [20]	RCT	Italien	Unsupervisiert	35	2024
Atoui et al. [1]	RCT	Kanada	Unsupervisiert	43	2024
Groen et al. [13]	Kohortenstudie	Niederlande	Klinisch	49	2024
Coca-Martinez et al. [10]	RCT	Kanada/Spanien	Klinisch + Zuhause	27	2024
Bojesen et al. [7]	RCT	Dänemark	Klinisch	16	2023
Molenaar et al. [17]	RCT	International	Klinisch	123	2023
Heil et al. [14]	Kohortenstudie	Niederlande	Klinisch	123	2023
Waller et al. [24]	RCT	Großbritannien	Zuhause	11	2022
Suen et al. [22]	Pilotintervention	Australien	Klinisch	50	2022
Fulop et al. [11]	RCT	Ungarn	Klinisch + Zuhause	77	2021
Carli et al. [9]	RCT	Kanada	Klinisch + Zuhause	55	2020
Bousquet-Dion et al. [8]	RCT	Kanada	Zuhause	37	2018

Auffällig ist zudem, dass die Prähabilitationsprogramme in den Studien in verschiedenen Settings durchgeführt wurden. Während einige RCTs rein klinikbasierte Programme untersucht haben, die eine engmaschige Betreuung durch interdisziplinäre Teams ermöglichen, setzen andere auf digitale oder hybride Konzepte. So wurden Home-based-Programme evaluiert, bei denen Patient:innen unter Anleitung von Onlineplattformen [4, 12, 13] oder mit sporadischer Supervision durch Fachpersonal trainieren konnten. Andere Studien haben eine Mischung aus stationären und ambulanten Komponenten implementiert, um eine flexiblere Betreuung zu ermöglichen.

Bereits existierende Best-Practice-Modelle für die Prähabilitation zeigen eindrucksvoll, dass eine systematische Implementierung dieser Programme sowohl klinische als auch wirtschaftliche Vorteile mit sich bringen kann. Besonders hervorzuheben ist das Universitätsklinikum Barcelona, das eine der längsten Erfahrungen mit einem routinemäßigen Prähabilitationsprogramm vorweisen kann [23]. Dort wurde ein multimodales Prähabilitationsprogramm etabliert, dass nicht nur die Effektivität hinsichtlich der Reduktion postoperativer Komplikationen und einer verbesserten funktionellen Erholung nachweisen konnte, sondern auch dessen Kosteneffizienz für das Gesundheitssystem belegen konnte [5, 6].

## Herausforderungen innerhalb und außerhalb von Zentren

Die Implementierung eines Prähabilitationsprogramms ist ein komplexer Prozess, der zahlreiche logistische Herausforderungen mit sich bringt. Damit solche Programme langfristig tragfähig und effizient in die klinische Praxis integriert werden können, müssen verschiedene Faktoren berücksichtigt werden.

### Multidisziplinäres Team, adäquate Infrastruktur und Ausstattung

Prähabilitationsprogramme umfassen verschiedene medizinische Fachbereiche und sollten daher interdisziplinär organisiert werden. Eine enge Zusammenarbeit zwischen Chirurgie, Anästhesie, Physiotherapie, Ernährungsberatung, Psychologie und Pflegepersonal ist essenziell. Darüber hinaus ist eine geeignete Infrastruktur erforderlich, die zumindest Untersuchungsräume für Konsultationen sowie ausreichend Platz und Equipment für körperliches Training umfasst [9].

### Modulare und flexible Programmgestaltung

Ein einheitliches Prähabilitationskonzept eignet sich nicht für alle Patient:innen gleichermaßen. Stattdessen sollte ein modulares System etabliert werden, das es ermöglicht, individuell angepasste Interventionen bereitzustellen. Patienten mit Mangelernährung, Sarkopenie oder ausgeprägter Gebrechlichkeit benötigen möglicherweise längere Vorbereitungszeiten [16, 21].

### Patientenmotivation und -einbindung

Die aktive Beteiligung von Patient:innen und deren Angehörigen ist entscheidend für den Erfolg eines Prähabilitationsprogramms. Die präoperative Phase stellt ein „window of opportunity“ dar, in dem Patient:innen durch die Aussicht auf eine verbesserte Genesung eher bereit sind, ihren Lebensstil kurzfristig zu ändern. Dennoch kann es zu hohen Abbruchraten kommen, weshalb es wichtig ist, Faktoren zu identifizieren, die die Adhärenz beeinflussen. Hürden wie die Notwendigkeit eines Betreuers oder lange Anfahrtswege erschweren die Teilnahme an Präsenzsitzungen. Studien zeigen, dass solche organisatorischen Barrieren häufig die Hauptgründe für eine geringe Teilnahmequote sind.

### Übertragung in den ambulanten Bereich, Monitoring und digitale Unterstützung

Die Kapazitäten stationärer Prähabilitationsprogramme sind, vor allem durch die fehlende bzw. komplexe Bezahlung durch das Gesundheitssystem mehr als begrenzt. Daher sind einfache, außerhalb des Krankenhauses durchgeführte Interventionen – wie eine Steigerung der täglichen körperlichen Aktivität – von höchster Bedeutung (siehe auch Beitag von Wiskemann und Köppel in dieser Ausgabe von *Die Chirurgie*). Der Erfolg solcher Programme hängt jedoch maßgeblich von der Motivation der Patient:innen ab. Daher sollte zumindest ein Mindestmaß an Supervision gewährleistet sein [3, 4, 12].

Eine mögliche Lösung besteht in der teilweisen Übertragung des Programms in die ambulante Versorgung, etwa durch Kooperationen mit Sporteinrichtungen oder durch die Möglichkeit, Übungen im häuslichen Umfeld durchzuführen. Dadurch könnten mehr Patient:innen erreicht werden, ohne die stationären Ressourcen zu überlasten.

### Kontinuierliche Evaluation und Qualitätskontrolle

Um die Effektivität eines Prähabilitationsprogramms langfristig sicherzustellen, sind regelmäßige interne Audits und Erfolgskontrollen erforderlich. Die Standardisierung von Programmen auf Basis individueller Risikoprofile und die Identifikation von Faktoren, die den Erfolg der Prähabilitation beeinflussen, sind wesentliche Herausforderungen der kommenden Jahre.

Ländliche Regionen stehen vor mehreren Hürden bei der Umsetzung von Prähabilitationsprogrammen. Eine der größten Herausforderungen ist die begrenzte Infrastruktur. Im Gegensatz zu städtischen Ballungszentren verfügen ländliche Gebiete oft nicht über spezialisierte Kliniken und Fachpersonal. Die geografischen Distanzen zwischen Patient:innen und den nächsten Gesundheitsdienstleistern erschweren es, regelmäßige prähabilitative Maßnahmen durchzuführen. Hinzu kommen strukturelle Defizite in der medizinischen Versorgung, wie ein Mangel an Fachärzt:innen, Physiotherapeut:innen und Ernährungsberater:innen.

Problem der Prähabilitation in ländlichen Regionen ist die begrenzte Infrastruktur

Fehlende Mobilitätsoptionen, längere Anfahrtswege zu den wenigen verfügbaren Therapiezentren und eine geringere digitale Infrastruktur machen es schwierig, moderne telemedizinische oder hybride Prähabilitationsansätze zu nutzen. Digitale Lösungen wie Telemedizin oder onlinegestützte Trainingsprogramme (siehe Beitrag von Flemming in dieser Ausgabe von *Die Chirurgie*) könnten eine Alternative darstellen, sind aber oft durch schlechte Internetanbindung oder eine geringe digitale Gesundheitskompetenz der Patient:innen limitiert.

### Finanzierung

Ein zentraler Faktor, der die flächendeckende Umsetzung von Prähabilitationsprogrammen zusätzlich erschwert, ist die fehlende finanzielle Vergütung innerhalb des bestehenden Gesundheitssystems in Deutschland. Anders als die postoperative Rehabilitation, die klar im DRG(„diagnosis related groups“)-System verankert ist, gibt es für prähabilitative Maßnahmen bislang keine gesicherte Abrechnungsmöglichkeit. Dies führt dazu, dass Kliniken und niedergelassene Ärzt:innen kaum Anreize haben, entsprechende Programme außerhalb von Forschungsprojekten anzubieten. Gleichzeitig ist die strukturierte, patientenzentrierte Prähabilitation vergleichbar personalintensiv wie die Rehabilitation.

Zudem werden die bisher durchgeführten Studien nahezu ausschließlich durch Drittmittelförderung finanziert. Das bedeutet, dass prähabilitative Programme aktuell fast nur in der vorhandenen Infrastruktur universitärer Zentren realisiert werden können, da dort die notwendigen Ressourcen und personellen Kapazitäten durch Forschungsförderungen bereitgestellt werden. Eine nachhaltige Implementierung in die Regelversorgung ist jedoch ohne eine systematische Einbindung in bestehende Finanzierungsmodelle kaum möglich.

Dies hat zur Folge, dass Prähabilitationsprogramme oft nur temporär im Rahmen klinischer Studien existieren und nach Beendigung der Förderperiode nicht weitergeführt werden. Langfristige Kohortenstudien oder Versorgungsanalysen zur Erhebung belastbarer Registerdaten fehlen ebenfalls, was wiederum eine evidenzbasierte Argumentation für eine breitere Implementierung erschwert.

## Lösungsansätze für die Prähabilitation außerhalb von Zentren

Trotz der Herausforderungen gibt es Wege, wie Prähabilitation auch in ländlichen Regionen implementiert werden kann. Eine Lösung könnte die Dezentralisierung sein, bei der lokale Gesundheitseinrichtungen wie Arztpraxen, Apotheken oder Rehabilitationszentren einbezogen werden, um prähabilitative Maßnahmen wohnortnah anzubieten. Gerade die immer häufiger angewendete ambulante Rehabilitation könnte ein gutes infrastrukturelles Einfallstor der Prähabilitation sein. Auch der Einsatz von Telemedizin spielt hier eine wichtige Rolle. Über digitale Plattformen können Patienten in ländlichen Regionen von Spezialisten betreut werden, ohne dass sie lange Anfahrtswege in Kauf nehmen müssen. Telemedizin ermöglicht zudem die Durchführung von Video-Therapiesitzungen und die Überwachung der Fortschritte der Patienten aus der Ferne [2–4].

## Prähabilitation – über spezialisierte Zentren hinausdenken

Prähabilitation hat sich in spezialisierten Zentren als effektives Konzept zur Reduktion postoperativer Komplikationen und zur Verbesserung der funktionellen Erholung etabliert. Doch die Implementierung darf nicht auf universitäre Einrichtungen beschränkt bleiben. Vielmehr müssen Möglichkeiten geschaffen werden, prähabilitative Maßnahmen in die Regelversorgung zu integrieren, sodass sie unabhängig von der Krankenhausstruktur oder dem Standort für eine breitere Patientengruppe zugänglich werden.

Internationale Best-Practice-Modelle zeigen, dass eine flächendeckende Implementierung gelingen kann, wenn innovative Ansätze genutzt werden. Neben klassischen, klinikbasierten Programmen gibt es zunehmend hybride oder digital unterstützte Konzepte, die eine dezentrale Durchführung ermöglichen. Telemedizinische Lösungen, Heimtrainingsprogramme mit digitaler Supervision und sektorübergreifende Versorgungsnetzwerke haben das Potenzial, Prähabilitation auch in nichtuniversitären Kliniken oder ambulanten Strukturen zu etablieren.

Die Umsetzung solcher Programme erfordert eine enge Vernetzung zwischen spezialisierten Zentren und wohnortnahen Gesundheitseinrichtungen. Hausärzt:innen, Physiotherapiepraxen, Apotheken und Rehabilitationszentren könnten stärker in prähabilitative Maßnahmen eingebunden werden, um eine wohnortnahe Betreuung sicherzustellen. Eine gezielte Schulung des Fachpersonals und die Schaffung verbindlicher Qualitätsstandards sind dabei essenziell.

Eine präoperative Patientenoptimierung braucht entsprechende Abrechnungsmöglichkeiten

Neben strukturellen Anpassungen ist auch eine politische und finanzielle Unterstützung erforderlich, um die Prähabilitation in die Regelversorgung zu überführen. Während die postoperative Rehabilitation über bestehende Vergütungssysteme abgedeckt ist, fehlt es für die präoperative Optimierung bislang an entsprechenden Abrechnungsmöglichkeiten. Eine Integration in bestehende Programme wie das Patient Blood Management (PBM) oder ERAS®-Konzepte könnte eine nachhaltige Finanzierung erleichtern.

Um eine prähabilitative Versorgung über spezialisierte Zentren hinaus zu gewährleisten, sind folgende Handlungsempfehlungen zentral:

- Ausbau telemedizinischer Angebote zur digitalen Supervision von Heimprogrammen (siehe Beitrag von Flemming in dieser Ausgabe von *Die Chirurgie*),
- Förderung sektorübergreifender Gesundheitsnetzwerke, die eine prähabilitative Betreuung auch außerhalb großer Kliniken ermöglichen (siehe Artikel von Wiskemann und Köppel in dieser Ausgabe von *Die Chirurgie*),
- Schaffung finanzieller Anreize für medizinisches Personal, um prähabilitative Maßnahmen in den ambulanten Bereich zu integrieren,
- Etablierung von Registerstudien, um belastbare Evidenz für die Versorgungsrealität jenseits universitärer Zentren zu generieren,
- verbindliche Standards in Bezug auf den Inhalt einer Prähabilitation (siehe Beitrag von Braun et al. und Detemble und Schnitzbauer in dieser Ausgabe von *Die Chirurgie*)

Die bisherigen Best-Practice-Modelle haben gezeigt, dass Prähabilitation nicht zwingend an spezialisierte Zentren gebunden sein muss. Die Herausforderung besteht nun darin, die gewonnenen Erkenntnisse auf breiter Ebene umzusetzen, um die präoperative Optimierung als festen Bestandteil der chirurgischen Versorgung zu etablieren.

## Fazit für die Praxis

- *Prähabilitation als Standard etablieren*: Zunehmende Evidenz und Leitlinienintegration unterstreichen die Notwendigkeit, Prähabilitation als festen Bestandteil der perioperativen Versorgung zu verankern.
- *Über Zentren hinausdenken*: Die Implementierung sollte nicht auf spezialisierte Kliniken beschränkt bleiben, sondern auch in die ambulante Versorgung integriert werden.
- *Technologie nutzen*: Telemedizin, hybride Modelle und digitale Supervision können die Prähabilitation auch außerhalb großer Kliniken ermöglichen.
- *Finanzierung sichern*: Eine nachhaltige Vergütung im Gesundheitssystem ist essenziell, um Prähabilitationsprogramme langfristig wirtschaftlich tragfähig zu machen.
- *Evidenz generieren*: Langfristige Registerstudien und Qualitätskontrollen sind notwendig, um die Wirksamkeit und Effizienz der Prähabilitation in der breiten Versorgung nachzuweisen.
